# Inadequate fine needle aspiration biopsy samples: Pathologists versus other specialists

**DOI:** 10.4103/1742-6413.52831

**Published:** 2009-06-18

**Authors:** GS Gomez-Macías, R Garza-Guajardo, J Segura-Luna, O Barboza-Quintana

**Affiliations:** * Department of Pathology and Cytopathology, Monterrey, Nuevo Leon, Mexico. Madero y Gonzalitos S/N Col. Mitras centro, Monterrey, N.L.,México. 64460; 1 Department of Investigation of the Autonomous University of Nuevo Leon, Monterrey, Nuevo Leon, Mexico. Madero y Gonzalitos S/N Col. Mitras centro, Monterrey, N.L.,México. 64460

**Keywords:** Fine needle aspiration biopsy, adequate specimens, influence of training, pathologist, other specialists

## Abstract

**Background:**

Fine needle aspiration biopsy (FNAB) is a simple, sensitive, quick and inexpensive method in which operator experience is essential for obtaining the best results.

**Methods:**

A descriptive study in which the aspiration biopsy cases of the Pathology and Cytopathology Service of the University Hospital of the UANL (2003–2005) were analyzed. These were divided into three study groups: Group 1, FNAB performed by a pathologist; Group 2, FNAB performed by specialists who are not pathologists, Group 3, FNAB guided by an imaging study with immediate evaluation by a pathologist. The samples were classified as adequate and inadequate for diagnosis, the organ, the size and characteristics of the lesions were taken into consideration.

**Results:**

A total of 1905 FNAB were included. In Group 1: 1347 were performed of which 1242 (92.2%) were adequate and 105 (7.7%) were inadequate. Of the 237 from Group 2, 178 were adequate (75.1%) and 59 inadequate (24.8%); in Group 3 there were 321 of which 283 (88.1%) were adequate and 38 (11.8%) inadequate. A statistically significant difference was found between FNAB performed by Group 1 (p< 0.001) and the other groups. A multivariate analysis was done where the organ punctured, the study groups, the size and characteristics of the lesion by study group were compared, finding that the most important variable was the person who performed the procedure.

**Conclusion:**

The experience and training of the person performing the aspiration biopsy, as well as immediate evaluation of the material when it is guided, substantially reduces the number of inadequate samples, improving the sensitivity of the method as well as reducing the need for open biopsies to reach a diagnosis.

## INTRODUCTION

Fine needle aspiration biopsy (FNAB) is a quick, sensitive and inexpensive technique for diagnosing benign and malignant palpable lesions[1] and non-palpable lesions from which material can be obtained with image-guidance.[2] The greatest advantage of this minimally invasive technique is its high sensitivity and specificity.[3] As a result, it is often utilized in the initial diagnostic evaluation of lesions identified in the breast,[4] thyroid[5] lymph nodes[6] and other organs.

The lower cost-benefit ratio of FNAB over other traditional biopsy types has been well documented. In addition, FNAB can be accurately performed as an outpatient procedure with fewer complications than an open biopsy.[7] The experience and training of the physician performing the FNAB has been shown to be a key factor in the quality and adequacy of the FNAB specimen. Yusef *et al*. showed that, when a physician lacked training, the rate of inadequate specimens for diagnosis was up to 29.5% in contrast to 4.6% when the sample is obtained by an appropriately trained pathologist.[8]

On the other hand, immediate cytologic evaluation of aspiration specimens obtained by an interventional radiologist has been shown to be crucial for the success of this procedure.[9] Immediate cytologic evaluation is also important when FNAB are performed by other specialists using imaging methods, such as endoscopic ultrasound-guided FNAB.[10]

This study evaluated the rates of inadequate material for diagnosis based on the physician performing the procedure, the importance of immediate cytologic assessment of samples obtained by image-guided aspiration, the influence of organ site and the size and characteristics of the lesions aspirated.

## MATERIALS AND METHODS

This descriptive retrospective study was performed in the Universidad Autónoma de Nuevo León Pathology and Cytopathology Laboratories of the University Hospital. The data were obtained by reviewing the laboratory database and cytology reports of the FNABs performed between January 2003 and December 2005. The FNABs were divided into three study groups: Group 1: FNABs performed by pathologists and/or residents under their supervision; Group 2: FNABs performed by non-pathology specialists, and Group 3: Image-Guided FNABs with immediate cytologic evaluation by a pathologist at the time of aspiration.

The FNABs in the three study groups were performed using a 10 ml plastic syringe with a 22-gauge needle; Group 1 also used a Cameco syringe pistol. The Group 1 FNABs consisted of four smears from each aspiration and a maximum of three punctures per procedure; two smears were fixed in alcohol and stained with diff quick stain for immediate pathologist evaluation. The other two smears were fixed in alcohol and stained using the Papanicolaou technique. The Group 1 FNABs were performed by pathologists, pathology residents and occasionally by residents from other specialties (surgery and gynecology) under a pathologist's supervision. In Group 2, the FNAB procedure, smear preparation, and alcohol fixation were carried out by other specialists who are not pathologists and subsequently sent to the pathology service. No immediate cytologic evaluation was performed during these procedures. These FNABs had an average of 2 to 4 slides prepared from an unknown number of punctures. All the slides in this group were stained with the Papanicolaou technique and interpreted by a pathologist. In Group 3, the FNAB was performed by a radiologist with smear preparation and fixation done by a pathology resident, who was present during the procedure for immediate evaluation. The smears were stained with the Papanicolaou technique. An average of three punctures with 4 to 6 slides were prepared per procedure.

The same adequacy criteria were used for all three study groups. Samples were considered adequate for interpretation when they contained a minimum of three well-preserved cell groups per slide and no fixation artifacts in the thin smears. All slides that were classified as inadequate for interpretation and 50% of the adequate slides were reevaluated in a blinded fashion by two cytopathologists using the aforementioned criteria.

The results were analyzed by study groups, organ sites punctured, and grouped by frequency.

**Figure 1:**
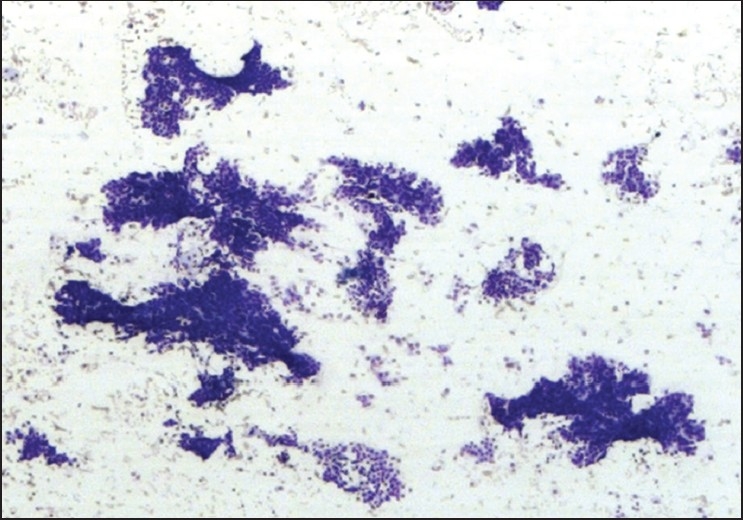
Adequate specimen

**Figure 2:**
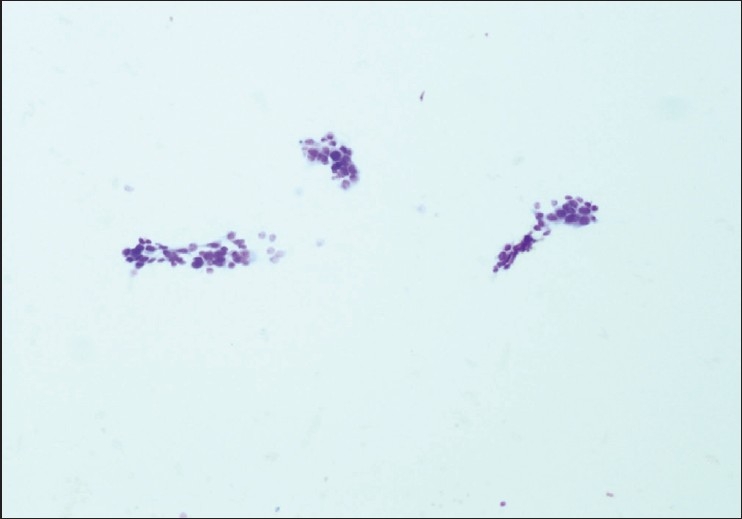
Inadequate specimen

The FNAB specimens were classified as adequate [Figure 1] or inadequate [Figure 2] for diagnosis. A comparative analysis of the three study groups was done using the X^2^ statistical test. Also, multivariate analysis was performed using the primary study groupings, organ sites punctured, the lesion sizes (less than 1cm, 1 to 3 cm and more than 3 cm), and the characteristics of the lesion, using the Kruskal-Wallis test. When less than five specimens were present in a subgroup, the Jonckheere-Terpstra test was used. A *p* value of <0.05 was considered to be statistically significant.

## RESULTS

1905 FNABs were identified for study and divided into the three primary study groups as follows: Group 1 n=1347 (70.7%), Group 2 n=237 (12.4%), and Group 3 n=321 (16.9%). The original diagnosis was confirmed in all of the reevaluated cases. In Group 1, 1242 (92.3%) of the FNABs were adequate for interpretation and 105 (7.7%) were inadequate. In Group 2, 178 (75.2%) were adequate and 59 (24.8%) were inadequate. In Group 3, 283 (88.2%) were adequate and 38 (11.8%) were inadequate. Group 1 contained a statistically higher percentage of adequate FNABs that the other two groups (*p*<0.001) [Table 1].

**Table 1:** Results according to study group

	*Adequate*	%	*Inadequate*	%	*Total*
Group 1 (Pathologists)	1242	92.2	105	7.7	1347
Group 2 (Other physicians)	178	75.1	59	24.8	237
Group 3 (Image-guided)	283	88.1	38	11.8	321

*P* = 0.001 in favor of group 1

The frequency of adequate and inadequate FNAB samples from each organ site is presented in Table 2. When subdivided by organ site, the Group 1 FNABs generally showed higher rates of adequacy.

**Table 2:** Comparsion of punctured organs

	*Carried out by pathologists*				*Image-guided*				*External*				*Total*	*P*
					
	*A*	*%*	*I*	*%*	*A*	*%*	*I*	*%*	*A*	*%*	*I*	*%*		
Breast	425	94.4	25	5.5	22	91.6	2	8.3	49	72	19	27.9	542	< 0.05
Thyroid	166	89.2	20	10.7	26	81.2	6	18.7	38	67.8	18	32.1	274	< 0.01
Lymph node	288	90.2	31	9.7	18	85.7	3	14.2	17	85	3	15	360	NS
Soft tissue	266	92.6	21	7.3	6	100	0	0	45	83.3	9	16.6	347	NS
Others	97	92.3	8	7.6	4	100	0	0	29	74.3	10	25.6	148	NA
Total	1242	92.2	105	7.7	76	87.3	11	12.6	178	75.1	59	24.8	1671	NA

A= Adequate, I = Inadequate, NS= Not significant, NA = Not analyzed

The organ site with the greatest number of FNABs was the breast with a total of 542 procedures. Group 1 performed 450 of the breast FNABs with 94.5% adequacy and 5.5% inadequacy rates. In comparison, Group 2 performed 68 FNABs with 72.0% being adequate and 28.0% inadequate for interpretation. Group 3 performed 24 FNABs with 91.6% being adequate and 8.4% inadequate for interpretation. Group 1 contained a statistically higher percentage of adequate FNABs than the other two groups (*p*<0.05).

The second most frequent organ site was from the lymph nodes with 360 cases. Group 1 performed 319 of these FNABs with 90.3% being adequate and 9.7% inadequate for interpretation. Group 2 carried out 20 of these FNABs with 85.0% being adequate and 15.0% inadequate. Group 3 performed 21 of these FNABs with 85.7% being adequate and 14.3% inadequate. In this subgroup, no significant difference was identified.

Soft tissue FNABs were the third most common procedure with a total of 347 specimens. Group 1 performed 287 of these FNABs with 92.6% being adequate and 7.4% inadequate samples. Group 2 performed 54 FNABS with 83.4% being adequate and 16.6% inadequate. In Group 3, six FNABs were performed with all being adequate. In this subgroup, no significant differences were identified.

The fourth organ in frequency was the thyroid gland with 274 punctures. Group 1 performed 186 of the FNABs with 89.2% being adequate and 10.8% inadequate. Group 2 carried out 56 FNABs with 67.8% being adequate samples and 32.2% inadequate samples. Group 1 contained a statistically higher percentage of adequate FNABs that the other two groups (*p*<0.01).

Deep organs such as the pancreas, liver, lung and others were biopsied using imaging techniques with pathology processing the specimen and providing immediate adequacy assessment of the material obtained; a total of 207 cases were performed of which 88.5% were adequate and 11.5% inadequate for interpretation [Table 3].

**Table 3:** Deep organ puncture/image-guided

*Organ*	*Adequate*	%	*Inadequate*	%	*Total*
Pancreas					
Biliary tree	51	89.4	6	10.5	57
Liver	75	91.4	7	8.5	82
Mediastinum	23	82.1	5	17.8	28
Lung	32	86.4	5	13.5	37
Kidney	26	86.6	4	13.3	30
Total	207	88.4	27	11.5	234

When comparing the lesion's size with specimen adequacy for each organ group, Group 1 generally showed statistically higher rates of adequacy, regardless of the lesion's size (less than 1 cm, 1 to 3 cm and more than 3 cm) (*p*<0.001) [Table 4]. With regard to the smaller number of inadequate samples, a statistical Group 1 advantage was not documented for soft tissue lesions less than 1 cm or with lesions greater than 3 cm in the breast and lymph nodes. In these latter groups, the number of cases was small [Table 5].

**Table 4:** Comparison of size of adequate lesions by organ punctured and study group

*Organ*	*Study group*	*Less than 1 cm (Adequate)*			*1 to 3 cm (Adequate)*			*More than 3 cm (Adequate)*			*Total*
					
		*A*	*%*	*P*	*A*	*%*	*P*	*A*	*%*	*P*
	Group 1	55	82		350	97.2		20	86.9		425
Breast	Group 2	3	50	*0.000	43	75.4	*0.000	3	60	*0.000	49	
	Group 3	8	88.8		14	93.3		0	0		22
Thyroid	Group 1	29	87.8		130	89.6		7	87.5		166	
	Group 2	3	75	*0.000	34	68	*0.000	1	50	*0.000	38	
	Group 3	8	80		18	81.8		0	0		26	
	Group 1	62	84.9		218	92.3		8	80		288	
Lymph node	Group 2	1	50	*0.000	15	93.7	*0.000	1	50	*0.000	17	
	Group 3	5	71.4		12	92.3		1	100		18	
	Group 1	19	86.3		65	87.3		182	95.2		266	
Soft tissues	Group 2	3	75	*0.000	10	83.3	*0.000	32	84.2	*0.000	45	
	Group 3	3	100		2	100		1	100		6	
	Group 1	15	88.2		68	94.4		14	87.5		97	
Others	Group 2	1	25	*0.000	20	90.9	*0.000	8	61.5	*0.000	29	
	Group 3	1	100		3	100		0	0		4
Total		216	82.4		1002	91.1		278	89.9		1496

* *P* in favor of group 1. A=Adequate

**Table 5:** Comparison of size of inadequate lesions by organ punctured and study group

*Organ*	*Study group*	*Less than 1 cm (Inadequate)*			*1 to 3 cm (Inadequate)*			*More than 3 cm (Inadequate)*			*Total*
					
		*I*	%	*P*	*I*	%	*P*	*I*	%	*P*	
	Group 1	12	17.9		10	2.7		3	13		25
Breast	Group 2	3	50	*0.000	14	24.5	*0.000	2	40	0.083 NS	19	
	Group 3	1	11.1		1	6.6		0	0		2
Thyroid	Group 1	4	12.1		15	10.3		1	12.5		20	
	Group 2	1	25	*0.046	16	32	*0.000	1	50	NA	18	
	Group 3	2	20		4	18.1		0	0		6	
	Group 1	11	15		18	7.6		2	20		31
Lymph node	Group 2	1	50	*0.000	1	6.25	*0.000	1	50	0.157 NS	3	
	Group 3	2	28.5		1	7.6		0	0		3	
	Group 1	3	13.6		9	12.1		9	4.7		21
Soft tissues	Group 2	1	25	0.083 NS	2	16.6	*0.005	6	15.7	*0.001	9	
	Group 3	0	0		0	0		0	0		0	
	Group 1	2	11.7		4	5.5		2	12.5		8
Others	Group 2	3	75	*0.046	2	9	*0.025	5	38.4	*0.014	10	
	Group 3	0	0		0	0		0	0		0
Total		46	17.5		97	8.8		32	10		175

* *P* in favor of group 1. I = Inadequate. NS = Not significant, NA = Not analyzed

The deep organs punctured by radiologists were also classified according to lesion size with most of the lesions biopsied in this group measuring 1 to 3 cm [Table 6].

**Table 6:** Size of lesions according to deep organ punctured/image-guided

*Organ*	*Less than 1 cm*	%	*E 1 to 3 cm*	%	*More than 3 cm*	%	*Total*
Pancreas and biliary tree	14	24.5	38	66.6	5	8.7	57
Liver	8	9.7	26	31.7	48	58.5	82
Mediastinum	0	0	7	25	21	75	28
Lung	9	24.3	20	54	8	21.6	37
Kidney	1	3.3	12	40	17	56.6	30
Total	32	13.6	103	44	99	42.3	234

With regards to the lesion's characteristics such as consistency (soft or firm), fibrosis, necrosis and cystic degeneration, the FNABs were compared by study group and organ site biopsied. Group 1 showed a statistical advantage (p< 0.001) in terms of adequate sample acquisition and lower numbers of inadequate samples [Tables 7 and 8].

**Table 7:** Comparison of adequate lesion characteristics by group

*Characteristics of the lesions*	*Group 1 adequate (N=1347)*		*Group 2 adequate (N=237)*		*Group 3 adequate (N=321)*		*Total*	*P*
			
	*A*	*%*	*A*	*%*	*A*	*%*		
Consistency								
Soft	257	19.0	52	21.9	103	32.0	412	(SC) *0.000
Firm	985	73.1	126	53.1	174	54.2	1285	(FC) *0.000
Presence of fibrosis	226	16.7	32	13.5	25	7.7	283	*0.000
Presence of necrosis	195	14.4	27	11.3	61	19.0	283	*0.000
Cystic degeneration	168	12.4	28	11.8	33	10.2	229	*0.000

* *P* in favor of group 1; (SC) = Soft consistency; (FC) = Firm consistency; A = Adequate

**Table 8:** Comparison of inadequate lesion characteristics by group

Characteristics of the lesions	*Group 1 Inadequate (n=1347)*		*Group 2 Inadequate (n=237)*		*Group 3 Inadequate (n=321)*		*Total*	*P*
					
	i	%	i	%	i	%		
Consistency								
Soft	40	2.9	11	4.6	18	5.6		
Firm	65	4.8	48	20.2	26	8.0		
Presence of fibrosis	95	7.0	14	5.9	11	3.4	120	*p= 0.000
Presence of necrosis	65	4.8	11	4.6	9	2.8	85	*p=0.000
Cystic degeneration	30	2.2	6	2.5	10	3.1	46	*p=0.000

* ***p*** in favor of group 1. SC = Soft consistency, FC = Firm consistency, I = Inadequate

With regard to the FNABs done by pathologists or pathology residents (pathology personnel) and residents from other specialties that rotate on the service, 1012 FNABs were performed by pathology personnel 94.0% being adequate and 6.0% being inadequate. A total of 335 FNABs were performed by other specialty residents that rotated on the pathology service with 87.0% being adequate and 13.0% being inadequate. The FNABs performed by pathology personnel contained a statistically significant higher percentage of adequate FNABs than the other group (*p*<0.01), which supports the premise that experience and adequate operator training in the procedure are directly proportional to the rate of obtaining adequate samples [Table 9].

**Table 9:** Physicians who carried out FNAB in the aspiration room

	*Adequate*	%	*Inadequate*	%	*Total*
Pathology personnel	952	94	60	5.9	1012
*Medico rotatorio	290	87.1	45	13.4	335

*P* < 0.01 in favor of t pathology personnel

* Other specialty residents that rotated on the pathology service

## DISCUSSION

FNAB is a currently accepted procedure for the initial diagnosis of patients with lesions when combined with clinical data and imaging. FNAB can be used to evaluate a number of diverse organ pathologies in the thyroid,[11–16] breast,[17–23] lymph node[24–27] and other organs. Its usefulness has increased in the diagnosis of deep organ lesions when combined with image guidance techniques such as conventional ultrasound, computerized tomography[28–29] or transendoscopic ultrasound.[30–32]

In addition to its high specificity and sensitivity [Table 10], FNAB has numerous advantages[33] such as lower cost, outpatient technique, minimally invasive approach and the possibility of obtaining material for special histochemical or immunohistochemical staining, electronic microscopy, flow cytometry and/or culture.[34] To use these adjuvant tests, it is essential that the cytologic material be adequate in both quality and quantity to make an accurate diagnosis. In the literature, reports of unsatisfactory or inadequate quantities of material varies from 3.0% to 14.0%.[8,35] These studies have linked the results to the knowledge and experience of the aspiration biopsy technique by the physician.

**Table 10:** Sensitivity and specificity of FNAB according to other studies

*Organ*	% *Sensitivity*	% *Specificity*
Breast 4	98	97
Lymph node 25	90	82.3
Thyroid 11	77.5	90.1
Diverse organs 3	93	100

Carson *et al.*[35] reviewed 2199 FNABs and found a significant difference in the percentage of inadequate material for interpretation cases between clinicians (14.0%) and pathologists (3.0%). Similarly, Yusef *et al.*[8] studied 692 FNABs and found that 29.5% were unsatisfactory samples when performed by surgeons in comparison with 4.6% when performed by pathologists. Other studies have described similar percentages.[3,35,36] In this study, other specialists, such as endocrinologists and gynecologists were included in addition to surgeons.

Ljung *et al.*[36] showed a notable difference in the results when FNAB is performed by physicians with training in the technique (2.0% inadequate material) versus physicians without training (25.0% inadequate material). The results of this study are similar with 7.7% inadequate specimens obtained in the aspiration clinic by trained pathology personnel versus 24.8% of specimens obtained by other specialists. The factors that contributed to this difference appeared to be mainly skill in the technique and the availability of immediate cytologic evaluation of the aspirate for adequacy. The latter assisted in determining if another organ aspiration was necessary and what specimens should be put aside for special studies. In addition, the pathologist performing the procedure has the advantages of being able to directly interact with the patient to obtain relevant clinical data and establish the characteristics of the lesion such as its exact location, size and consistency.[8,33]

Variables such as the lesion's size and characteristics did not appear to affect the rate of obtaining adequate samples when the FNAB was performed by pathology personnel in comparison with the other groups. Multivariate analysis indicated that adequate training, skill in the technique, and immediate cytologic evaluation carried more weight in predicting the success of obtaining an adequate sample.

The relationship between inadequate samples and the organ site showed that the breast had the lowest percentage of unsatisfactory samples by pathology personnel (5.5%), in contrast to 10.7% for the thyroid and 9.7% for lymph nodes [Table 2]. Yusef *et al.* in their study showed a greater percentage of non-diagnostic aspirates in breast tissue, followed by lymph node and thyroid.[8]

Multidisciplinary collaboration between a radiologist and a pathologist when performing image guided FNAB in deep organs is also fundamental, since recent studies have indicated that immediate cytologic evaluation of the sample by a pathologist influences specimen adequacy and improves the cost-benefit ratio.[37,38] In this study, ultrasound or computerized tomography guided FNABs that were initially evaluated by a pathologist had better results (11.8% inadequate samples) than those without an immediate cytologic evaluation by a pathologist (24.8% inadequate samples).

In contrast to other studies, a difference was identified between FNABs performed by pathology personnel (5.9% inadequate material) and residents from other specialties who were in training on the pathology service (13.4% inadequate material, p<0.01). These results support that more training time and supervision produces better results.

## CONCLUSION

Based on the results of this study, it is recommend that fine needle aspiration biopsy be performed preferably by a pathologist who has received specific training in this technique, who can carry out an immediate evaluation of the material, decide if it is necessary to re-aspirate and can distribute the aspirated material for ancillary studies. The aforementioned contrasts with the results obtained by other specialists where it is unknown if they have received specific FNAB training and knowledge of the aspiration smear technique. The results of this study support that it is essential to include aspiration biopsy techniques in pathology residency training under the direct supervision of an experienced cytopathologist.

On the other hand, it is important to emphasize that deep organ aspirations and lesions under 1 cm may be performed by interventional radiologists in concert with a pathologist to ensure optimal smearing, fixation, and immediate evaluation with the goal of lowering the rate of unsatisfactory results and increasing the cost-benefit of the procedure.

## COMPETING INTEREST STATEMENT BY ALL AUTHORS

No competing interest to declare by any of the authors

## AUTHORSHIP STATEMENT BY ALL AUTHORS

All authors of this article declare that we qualify for authorship as defined by ICMJE.

**Authorship credit:** GM substantial contributions to conception and design, acquisition of data, or analysis and interpretation of data; GG and BQ drafting the article or revising it critically for important intellectual content; SL performed the statistical analysis. GM, GG, BQ, and SL final approval of the version to be published.

Each author has participated sufficiently in the work and take public responsibility for appropriate portions of the content of this article.

## ETHICS STATEMENT BY ALL AUTHORS

This study was conducted with approval from Institutional Review Board (IRB) of all the institutions associated with this study. Authors take responsibility to maintain relevant documentation in this respect.

## References

[1] Frable WJ (1989). Needle aspiration biopsy: Past, present, and future. Hum Pathol.

[2] Chojniak R, Isberner RK, Viana LM, Yu LS, Aita AA, Soares FA (2006). Computed tomography guided needle biopsy: Experience from 1,300 procedures. Sao Paulo Med J.

[3] Florentine BD, Staymates B, Rabadi M, Barstis J, Black A (2006). Cancer Committee of the Henry Mayo Newhall Memorial Hospital. The reliability of fine-needle aspiration biopsy as the initial diagnostic procedure for palpable masses: A 4-year experience of 730 patients from a community hospital-based outpatient aspiration biopsy clinic. Cancer.

[4] Ariga R, Bloom K, Reddy VB, Kluskens L, Francescatti D, Dowlat K (2002). Fine-needle aspiration of clinically suspicious palpable breast masses with histopathologic correlation. Am J Surg.

[5] Yang J, Schnadig V, Logrono R, Wasserman PG (2007). Fine-needle aspiration of thyroid nodules: A study of 4703 patients with histologic and clinical correlations. Cancer.

[6] Gupta RK, Naran S, Lallu S, Fauck R (2003). The diagnostic value of fine needle aspiration cytology (FNAC) in the assessment of palpable supraclavicular lymph nodes: A study of 218 cases. Cytopathology.

[7] Stewart CJ, Coldewey J, Stewart IS (2002). Comparison of fine needle aspiration cytology and needle core biopsy in the diagnosis of radiologically detected abdominal lesions. J Clin Pathol.

[8] Al-Marzooq YM, Chopra R, Al-Bahrani AT, Younis M, Al-Mulhim AS, Al-Mommatten MI (2004). Comparison of specimen adequacy in fine-needle aspiration biopsies performed by surgeons and pathologists. Ann Saudi Med.

[9] Nasuti JF, Gupta PK, Baloch ZW (2002). Diagnostic value and cost-effectiveness of on-site evaluation of fine-needle aspiration specimens: Review of 5,688 cases. Diagn Cytopathol.

[10] Vander Noot MR, Eloubeidi MA, Chen VK, Eltoum I, Jhala D, Jhala N (2004). Diagnosis of gastrointestinal tract lesions by endoscopic ultrasound-guided fine-needle aspiration biopsy. Cancer.

[11] Berner A, Pradhan M, Jørgensen L, Heilo A, Grøholt KK (2004). Fine needle cytology of the thyroid gland. Tidsskr Nor Laegeforen.

[12] Redman R, Zalaznick H, Mazzaferri EL, Massoll NA (2006). The impact of assessing specimen adecuacy and number of needle passes for fine- needle aspiration biopsy of thyroid nodules. Thyroid.

[13] Goldstein RE, Netterville JL, Burkey B, Johnson JE (2002). Implications of follicular neoplasms, atypia, and lesions suspicious for malignancy diagnosed by fine-needle aspiration of thyroid nodules. Ann Surg.

[14] Gharib H (1994). Current evaluation of thyroid nodules. Trends Endocrinol Metab.

[15] Sclabas GM, Staerkel GA, Shapiro SE, Fornage BD, Sherman SI, Vassillopoulou-Sellin R (2003). Fine-needle aspiration of the thyroid and correlation with histopathology in a contemporary series of 240 patients. Am J Surg.

[16] O'Malley ME, Weir MM, Hahn PF, Misdraji J, Wood BJ, Mueller PR (2001). Us-guided fine-needle aspiration biopsy of thyroid nodules: Adequacy of cytologic material and without immediate cytologic analysis. Radiology.

[17] Saxe A, Phillips E, Orfanou P, Husain M (2001). Role of sample adequacy in fine needle aspiration biopsy of palpable breast lesions. Am J Surg.

[18] Howell LP, Gandour-Edwards R, Folkins K, Davis R, Yasmeen S, Afify A (2004). Adequacy evaluation of fine-needle aspiration biopsy in the breast health clinic setting. Cancer.

[19] Kanchanabat B, Kanchanapitak P, Thanapongsathorn W, Manomaiphiboon A (2000). Fine-needle aspiration cytology for diagnosis and management of palpable breast mass. Aust N Z J Surg.

[20] Howat AJ (1998). Uniform approach to breast fine needle aspiration biopsy. Acta Cytol.

[21] Costa MJ, Tadros T, Hilton G, Birdsong G (1993). Breast fine needle aspiration cytology: Utility as a screening tool for clinically palpable lesions. Acta Cytol.

[22] Cohen MB, Rodgers RP, Hales MS, Gonzales JM, Ljung BM, Beckstead JH (1987). Influence of training and experience in fine-needle aspiration biopsy of breast: Receiver operating characteristics curve analysis. Arch Pathol Lab Med.

[23] Houssami N, Ciatto S, Ambrogetti D, Catarzi S, Risso G, Bonardi R (2005). Florence-Sydney Breast Biopsy Study: Sensitivity of ultrasound-guided versus freehand fine needle biopsy of palpable breast cancer. Breast Cancer Res Treat.

[24] Malami SA (2007). Uses and limitations of fine needles aspiration cytology in the diagnostic work-up of patients with superficial lymphadenopathy. Nig Q J Hosp Med.

[25] Steel BL, Schwartz MR, Ramzy I (1995). Fine needle aspiration biopsy in the diagnosis of lymphadenopathy in 1,103 patients: Role, limitations and analysis of diagnostic pitfalls. Acta Cytol.

[26] Mostafa MG, Chiemchanya S, Srivannaboon S, Nitiyanant P (1997). Accuracy of fine needle aspiration cytology in the evaluation of peripheral lymphadenopathy. J Med Assoc Thai.

[27] Martins MR, Santos Gda C (2006). Fine-needle aspiration cytology in the diagnosis of superficial lymphadenopathy: A 5-year Brazilian experience. Diagn Cytopathol.

[28] Sheth S, Hamper UM, Stanley DB, Wheeler JH, Smith PA (1999). US guidance for thoracic biopsy: A valuable alternative to CT. Radiology.

[29] Diederich S, Padge B, Vossas U, Hake R, Eidt S (2006). Application of a single needle type for all image-guided biopsies: Results of 100 consecutive core biopsies in various organs using a novel tri-axial, end-cut needle. Cancer Imaging.

[30] Chhieng DC, Jhala D, Jhala N, Eltoum I, Chen VK, Vickers S (2002). Endoscopic ultrasound-guided fine-needle aspiration biopsy: A study of 103 cases. Cancer.

[31] Garza R, Barboza O, Garza A, Flores J, Ancer J (2005). Biopsia por aspiració;n con aguja fina guiada por ultrasonido endoscópico. Experiencia del Hospital Universitario, UANL. Rev Hosp Gral.

[32] Vander Noot MR, Eloubeidi MA, Chen VK, Eltoum I, Jhala D, Jhala N (2004). Diagnosis of gastrointestinal tract lesions by endoscopic ultrasound-guided fine-needle aspiration biopsy. Cancer.

[33] Dabbs DJ (1998). The surgical pathologist's approach to fine needle aspiration. Clin Lab Med.

[34] Granville LA, Laucirica R, Verstovsek G (2008). Clinical significance of cultures collected from fine-needle aspiration biopsy. Diagn Cytopathol.

[35] Carson HJ, Saint Martin GA, Castelli MJ, Gattuso P (1995). Unsatisfactory aspirates from fine- needle aspiration biopsies: A review. Diagn Cytopathol.

[36] Ljung BM, Drejet A, Chiampi N, Jeffrey J, Goodson WH, Chew K (2001). Diagnostic accuracy of fine-needle aspiration biopsy is determined by physician training in sampling technique. Cancer.

[37] Saleh HA, Khatib G (1996). Positive economic and diagnostic accuracy impacts of on-site evaluation of fine-needle aspiration biopsies by pathologists. Acta Cytol.

[38] Ghofrani M, Beckman D, Rimm DL (2006). The value of onsite adecuacy assessment of thyroid fine-needle aspirations is a function of operator experience. Cancer Cytopat.

